# “There should be one spot that you can go:” *BRCA* mutation carriers’ perspectives on cancer risk management and a hereditary cancer registry

**DOI:** 10.1007/s12687-023-00685-5

**Published:** 2023-10-21

**Authors:** J. Hynes, L. Dawson, M. Seal, J. Green, M. Woods, H. Etchegary

**Affiliations:** 1 Faculty of Medicine, Memorial University, Craig L. Dobbin Centre for Genetics, Rm 4M210, St. John’s, NL A1B 3V6 Canada; 2 Department Obstetrics and Gynecology, Faculty of Medicine, University of British Columbia, Vancouver, BC Canada; 3 Cancer Care Program, Eastern Regional Health Authority, St. John’s, NL Canada

**Keywords:** BRCA pathogenic variants, Breast cancer, Ovarian cancer, Hereditary breast and ovarian cancer syndrome, High-risk cancer care management, Inherited cancer registry, Patient-oriented research

## Abstract

Individuals who carry *BRCA1* or *BRCA2* pathogenic variants are recommended to have extensive cancer prevention screening and risk-reducing surgeries. Uptake of these recommendations is variable, and there remains room for improvement in the risk management of *BRCA* carriers. This paper explores female *BRCA* carriers’ experiences with the current model of care and their perspectives on (and interest in) an inherited cancer registry. Findings can inform the development of a dedicated high-risk screening and management program for these patients. Quantitative and qualitative data were gathered through a provincial descriptive survey and semi-structured qualitative interviews to assess *BRCA* carriers’ opinions toward risk management services in the province of Newfoundland and Labrador (NL), Canada. Survey (*n* = 69) and interview data (*n* = 15) revealed continuity and coordination challenges with the current system of care of high-risk individuals. Respondents suggested an inherited cancer registry would help identify high-risk individuals and provide a centralized system of risk management for identified carriers. Respondents identified concerns about the privacy of their registry data, including who could access it. Findings suggest *BRCA* carriers see great value in an inherited cancer registry. Specifically, participants noted it could provide a centralized system to help improve the coordination of burdensome, life-long risk management. Important patient concerns about protecting their privacy and their health data confidentiality must be addressed in patient and public information and informed consent documents about a registry.

**Supplementary Information:**

The online version contains supplementary material available at 10.1007/s12687-023-00685-5.

##  Introduction

*BRCA1* and *BRCA2* are tumor suppressor genes responsible for repairing DNA double-strand breaks during cell division (Scully 2000). Mutations in these genes result in a cancer predisposition syndrome which confers a 50–70% lifetime risk of breast cancer (BC) and 20–40% lifetime risk of ovarian cancer (OC) in affected individuals (Easton et al. 1995; King et al. 2003; Petrucelli, 2016). Guidelines exist for the optimal management of *BRCA* mutation carriers, with evidence that cancer rates and all-cause mortality are decreased through appropriate risk management (NCCN 2023).

The National Comprehensive Cancer Network (NCCN) recommends that individuals with *BRCA* pathogenic variants begin breast cancer screening with semi-annual clinical breast exams by age 25, annual breast MRI between ages 25 and 75, and annual mammography between ages 30 and 75 (NCCN 2023). Bilateral prophylactic mastectomy (BPM) is an alternative option to surveillance offered to female *BRCA* mutation carriers. Given the ineffectiveness of screening for ovarian cancer, prophylactic risk-reducing salpingo-oophorectomy (RRSO) is recommended between the ages of 35 and 40 for *BRCA1* carriers and between the ages of 40 and 45 for *BRCA2* carriers (NCCN 2023). RRSO confers an associated ovarian cancer risk reduction of 80–90% and a breast cancer risk reduction estimated at 50%, particularly in *BRCA2* carriers (NCCN 2023). While some research is exploring the efficacy of early salpingectomy and delayed oophorectomy (Gaba et al. 2021), RRSO remains the standard of care for *BRCA* carriers (Tindale et al. 2022).

A comprehensive clinical review of *BRCA* pathogenic variant carriers in the Canadian province of Newfoundland and Labrador (NL) (Hand et al. 2023) revealed that nearly 34% of *BRCA* patients were not fully adherent to screening and prevention guidelines. A total of 73.3% of eligible individuals had undergone RRSO, and 39% of eligible patients had received a BPM. Of patients eligible for breast screening, 61.4% had an MRI, and 61.6% had a mammogram within the recommended screening periods. These rates are consistent with research in other *BRCA* populations (Buchanan et al. 2017; Hanley et al. 2019; Metcalfe et al. 2019), confirming that there is room for improvement in *BRCA* carriers’ care in our jurisdiction and Canada-wide. Indeed, Tindale et al. 2022 assert that “New and more effective models of care could decrease the burden, morbidity, and mortality of hereditary cancer and expand system capacity to provide this preventative health care to Canadians in an equitable way (p.4633).”

In the Eastern Canadian province of NL, patients receive genetic counseling and testing for *BRCA* pathogenic variants through the Provincial Medical Genetics Program, located in the capital city of St. John’s. *BRCA* carriers are given a results letter to share with other family members and are usually referred to medical oncology, but there is no systematic ongoing support or follow-up in place for these very high-risk individuals. Instead, patients and their family doctors (if indeed they have one) are left to navigate the complex and life-long risk management of a cancer predisposition syndrome.

Evidence from other jurisdictions suggests that high-risk individuals’ effective clinical management is possible through a dedicated inherited cancer risk management service and registry. Previous research has demonstrated a reduction in colorectal cancer incidence and mortality through carriers’ participation in an inherited cancer program underpinned by a registry (Barrow et al. 2013). Long-term management of *BRCA* carriers through a dedicated inherited cancer service is cost-effective, improves referral rates to appropriate care providers, increases the uptake of preventive surgeries, and improves cancer risk management (Lobo et al. 2018; Pitchert et al. 2010; Tindale et al. 2022). Notably, very few studies have explored patients’ views on the value of coordinated, centralized, healthcare services for carriers of cancer mutations, nor their interest in being part of an inherited cancer registry. However, in a recent study with a small sample of mutation carriers, Meiser et al. (2022) reported positive and altruistic reactions to the establishment of a national genomics registry of inherited cancer predisposition syndromes.

Canada has no dedicated national registry of carriers of highly penetrant cancer pathogenic variants. Similarly, in our study setting of NL, there is currently no inherited cancer registry dedicated to these high-risk families’ care. Instead, care is provided through a very small group of specialists with a professional interest in inherited cancers. However, this is not a formal program and does not capture all high-risk patients in the province.

This study is part of a more extensive research program designed to provide proof of concept of the value of a provincially funded inherited cancer risk management service. This paper presents quantitative and qualitative data from a patient-oriented, psychosocial sub-study, including selected results from a descriptive survey and semi-structured qualitative interviews with female *BRCA* carriers in NL. Specifically, this paper focuses on *BRCA* mutation carriers’ perspectives on (and interest in) an inherited cancer registry and explores carriers’ experiences with the current care model and their views on the value of a centralized care service. Ultimately, this work is guided by the belief that we need to consider new strategic directions in the model of hereditary cancer care and prevention and incorporate patients’ needs and preferences into care models.

## Methods

### Patient-oriented study design

This study received ethics approval from the provincial health research ethics board (Reference # 2018.010). A provincial survey and qualitative interviews explored and measured female *BRCA* carriers’ experiences with genetic testing, managing their increased cancer risk (including prophylactic surgical decisions), challenges faced with the current model of care, impact their *BRCA* mutation has on their lives, and perspectives about the need for an inherited cancer registry. The study was co-designed with three patient partners to ground the project in patients’ perspectives and priority research areas and included patient-reported experiences and outcomes.

### Measurement of adherence variable

Participants’ level of adherence to risk management recommendations was defined according to clinical guidelines (NCCN 2023), clinical judgment of the physicians on the team, and prior review of medical records of the *BRCA* population in NL (Hand et al. 2023). Carriers were considered very adherent if they were aged 25 to 75 years and were both adherent to breast guidelines and had completed RRSO (if eligible). Individuals 25- to 75-year olds with no breast screening or BPM and no RRSO (if eligible) were considered non-adherent. Individuals were considered moderately adherent when they had followed at least one guideline recommendation, but not all. For BPM, eligibility criteria were females between 25 to 75 years of age with breast(s) at the time of genetic testing. For MRI and mammogram, eligibility was similar—females with breast(s) at time of data analysis and 25 to 75 years of age (MRI) and 30 to 75 years of age (mammogram). Females with ovaries at the time of genetic testing were considered eligible for RRSO if 35–75 years with *BRCA1* mutations and 40 to 75 years of age if *BRCA 2* mutation carriers.

#### BRCA carrier postal survey

The research team created the descriptive survey informed by the literature on *BRCA* mutation carriers, providers’ clinical experience, and the lived experience of patient partners. The survey contained several sections, including opinions on developing a centralized, coordinated-care service and registry for families with hereditary breast ovarian cancer, perceived adherence to screening and prevention guidelines, and satisfaction with current health services. All items were measured on a 5-point Likert scale from Strongly Agree (1) to Strongly Disagree (5), such that higher mean responses indicated greater disagreement with items. Survey items related to registries are presented in this study, but the full survey is included in the Supplementary information.

### Survey sampling and recruitment

All known female *BRCA* pathogenic carriers in the province over 18 years old and residing in NL were eligible to receive the survey. Records from the Provincial Medical Genetics Program (PMGP) were queried to obtain a dataset of all *BRCA* carriers up to the time of the *BRCA* clinical review (which was 2017; Hand et al. 2023). Clinicians on the study team identified all patients who had received a *BRCA* mutation-positive result reported through the program clinically (since 2006) and through research (1994–2006), including individuals who had obtained results through private or out-of-province testing. A total of 140 surveys were mailed with one subsequent follow-up phone call from a research nurse to ensure receipt and answer questions.

### Clinical data

As noted, a review of BRCA carriers medical records in NL up to 2017 (Hand et al. 2023) extracted screening and surgical data including mammograms and RRSO in order to categorize carriers’ level of adherence to recommended risk management guidelines. Prior cancer history was also extracted. Those surveys that had correctly been given an ID # at the time of mailout were added to the clinical data file, allowing the linking of survey data to adherence level.

Data was analyzed using SPSS Software 27.0. Descriptive statistics including counts and percentages, as well as means and standard deviations where appropriate, are reported for demographic and survey items.

#### Qualitative interviews

A semi-structured interview guide (Supplementary information) was followed to ensure similar content was encompassed across interviews while allowing for question-order flexibility and clarification through probing. Semi-structured interviews also allowed patients to raise additional ideas that came to mind during the conversation. The interview questions aimed to gain a full picture of patients’ experience as a *BRCA* carrier in NL and their opinions on a provincial inherited cancer registry. Questions were constructed by the research team, in discussion with patient partners to ensure priority areas were included. Questions probed patients about how they manage their care and any barriers that limit their ability to access care, their willingness to take part in an inherited registry and their perceived advantages and concerns, and the types of research that are important to them. Prompts were used when necessary to gather the most comprehensive account of *BRCA* carriers’ experiences.

### Sampling and recruitment

Inclusion criteria consisted of females over the age of 18 with a *BRCA1* or *BRCA2* pathogenic variant confirmed through the PMGP. Carriers who had been previously diagnosed with cancer, who currently had a cancer diagnosis, or who had never received a cancer diagnosis were able to participate in the study, which ensured narratives were representative of a range of cancer experiences and risk management recommendations.

Recruitment was conducted by physicians within patients’ circle of care. Purposeful sampling was used to ensure variability in the range of experience with inherited cancer, differing years since receiving carrier status, differing numbers of affected family members, varying ages, and residence in different areas of the island. The goal of purposeful sampling, as described by Sandelowski et al. (2000) is, “to obtain cases deemed information rich for the purpose of the study.”

Participants were invited to participate in either an individual interview (in person, by phone, or by email) or a focus group with other *BRCA* carriers. Providing participants with multiple study involvement options allowed them to take part in the way most comfortable and accessible to them. Discussing cancer history and risk can be distressing; individual interview options allowed a private discussion. Ultimately, all participants chose an individual interview, and no group discussions were conducted.

### Data collection and analysis

Interviews were audio-recorded and transcribed verbatim. The interview guide was used for all interviews; however, questions were not necessarily asked in the same order in each interview, and participants were encouraged to ask any questions or raise any other issues important to them. Thus, while the order of topics varied slightly, all content areas on the interview guide were covered in every interview. Interviews proceeded until no new ideas or themes were developed. Once data saturation was deemed complete, no further interviews were conducted.

Qualitative description (Sandelowski 2000, 2010; Chafe 2017) was used to summarize the data about carriers’ accounts of ongoing management of inherited cancer risk. This form of naturalistic inquiry aims to present the data in the participants’ language, resulting in a comprehensive summary of the event in question. It is particularly useful for research aiming to provide data to inform health services provision (Chafe 2017), a key goal of this study.

The process of analyzing the data began with the familiarization of the transcripts. After the first three interviews were transcribed, transcripts were reviewed by JH and HE together, identifying emerging initial ideas and potential codes. After interviews were completed, a full review of the transcripts began. Interviews were read independently by these team members, again making notes of potential codes and themes. Both team members then met to discuss surface-level ideas and potential themes and codes that became relevant throughout the familiarizing processes. The goal of these sessions was to ensure transcriptions were thoroughly read and discussed.

Once all interviews had been actively read and ideas initiated, formally generating the initial codes began. Transcripts were again read in their entirety by JH, this time with a more active goal of generating as many codes as possible. A complete list of each potential codes was kept, attempting to code for as many potential ideas as possible. After all transcriptions had been re-read and a full code list generated, several higher-order themes became clear by noting which ideas fit under the same general label. At this stage, a complete list of codes, with their hierarchical themes, began to develop. It was important at this stage not to remove potential codes because they were not popular throughout many transcripts and avoid reducing codes down too much, as this can result in nonspecific codes. After the full code and theme chart were generated, NVivo Software was used to code all interviews with the complete list of codes and themes. Through this rigorous coding process, the most relevant and important themes became apparent, and it was evident that thematic saturation had indeed occurred. JH and HE then met to review and finalize final themes before they were shared with the study team and patient partners.

### Reflexivity considerations

This study is part of a larger program of research led by a team of clinicians and researchers who strongly believe in the value of inherited cancer registries and that a new model of coordinated care is needed for families with hereditary cancers. It was important for analysts (HE, JH) to be aware of this motivation in the analysis of data. During analysis meetings, JH and HE would continuously assess findings to ensure themes and codes developed logically from the transcripts and to discuss alternative interpretations. And as noted, final codes and themes were reviewed by patient partners as a further check.

It was also important that the interviewer appears impartial and neutral so participants understood they could provide information in a confidential space. For example, it was shared with participants that the interviewer (JH) was not connected to a particular doctor. This often allowed them to open up and share their healthcare experiences candidly, without concern the interviewer was partial.

Finally, close attention to context was paid during the generation of initial codes and final themes. For example, if a participant disclosed that they did not know about their family’s history of cancer, it was important to think about the context in which this information was given. A participant may not know about their family’s history of cancer because there actually is not a strong family history or because the participant was not close with members of their family and did not have access to this information or because they had never asked their family about this information. As such, not knowing about family history of cancer might be coded as “lack of family cancer knowledge,” whereas in other considerations of context, this might be coded as “unmotivated to learn about *BRCA* and cancer risk.”

## Results

### Survey participants

In total, 69 surveys were returned (response rate 49%). At the time of survey mailing, an administrative/clerical error meant that not all respondent names were given a survey ID #; thus, not all respondents’ survey data could subsequently be linked with their corresponding clinical data (e.g., cancer history, compliance with risk management guidelines). Ultimately, 44 of the 69 returned surveys were identified and linked to patients’ clinical and demographic data. While descriptive statistics are provided for survey response items for the entire dataset (*n* = 69), demographic and clinical data are provided only for identified survey respondents (*n* = 44).

Identified survey respondents whose clinical information could be linked (*n* = 44) had a mean age of 56.9 ± 12.1 years old, and it had been on average 8.12 ± 3.19 years since they underwent genetic testing. Prevalence of *BRCA1* (*n* = 20) and *BRCA2* (*n* = 24) mutations was fairly balanced as were the percentages living in rural and urban communities (Table 1). Just over one-third had a personal history of breast cancer, while 13% had a personal history of ovarian cancer. Five individuals were considered not adherent to risk management guidelines (12%), 20% were considered somewhat adherent, and 68% were considered very adherent.

**Table 1:** Demographic and clinical data on identified survey respondents*

Variable	Identified survey respondents *N* (%)
*BRCA1* vs. *BRCA 2*	
*BRCA*1	20 (45%)
*BRCA*2	24 (55%)
Urban vs. rural	
Urban	23 (53%)
Rural	20 (47%)
Risk-reducing salpingo-oophorectomy	
Yes	31 (70%)
No	13 (30%)
Mammogram in the last 18 months	
Yes	17 (41%)
No	24 (59%)
Adherence level	
Very adherent	28 (68%)
Moderately adherent	8 (20 %)
Not adherent	5 (12 %)
Personal diagnosis of breast cancer	
Yes	15 (35%)
No	28 (65%)
Personal diagnosis of ovarian cancer	
Yes	4 (12%)
No	28 (88%)

*Data based on 44 linked surveys only; totals do not always add to 44 due to missing data in patients’ charts

### Qualitative interview respondents

Interviews ranged from 32 to 62 min and were 45 min on average. Demographic and clinical information for interview participants is included in Table 2. Two-thirds were living in an urban area, and most were over the age of 40. Almost one-third had a personal history of breast cancer; 60% were very adherent to risk management guidelines.

**Table 2:** Interview participants’ demographic information

Characteristic	*n* (%)
Age	
Under 40	8 (53%)
Over 40	7 (47%)
Residence	
Rural	5 (33%)
Urban	10 (67%)
*BRCA*1 vs. *BRCA*2	
*BRCA*1	10 (67%)
*BRCA*2	5 (33%)
Risk-reducing salpingo-oophorectomy	
Yes	5 (33%)
No	10 (67%)
Risk-reducing mastectomy	
Yes	1 (7%)
No	14 (93%)
Adherence level	
Not adherent	1 (7%)
Moderately adherent	5 (33%)
Very adherent	9 (60%)
Breast cancer	
Yes	4 (27%)
No	11 (73%)
Ovarian cancer	
Yes	1 (7%)
No	14 (93%)

### Survey findings

**Fig. 1:**
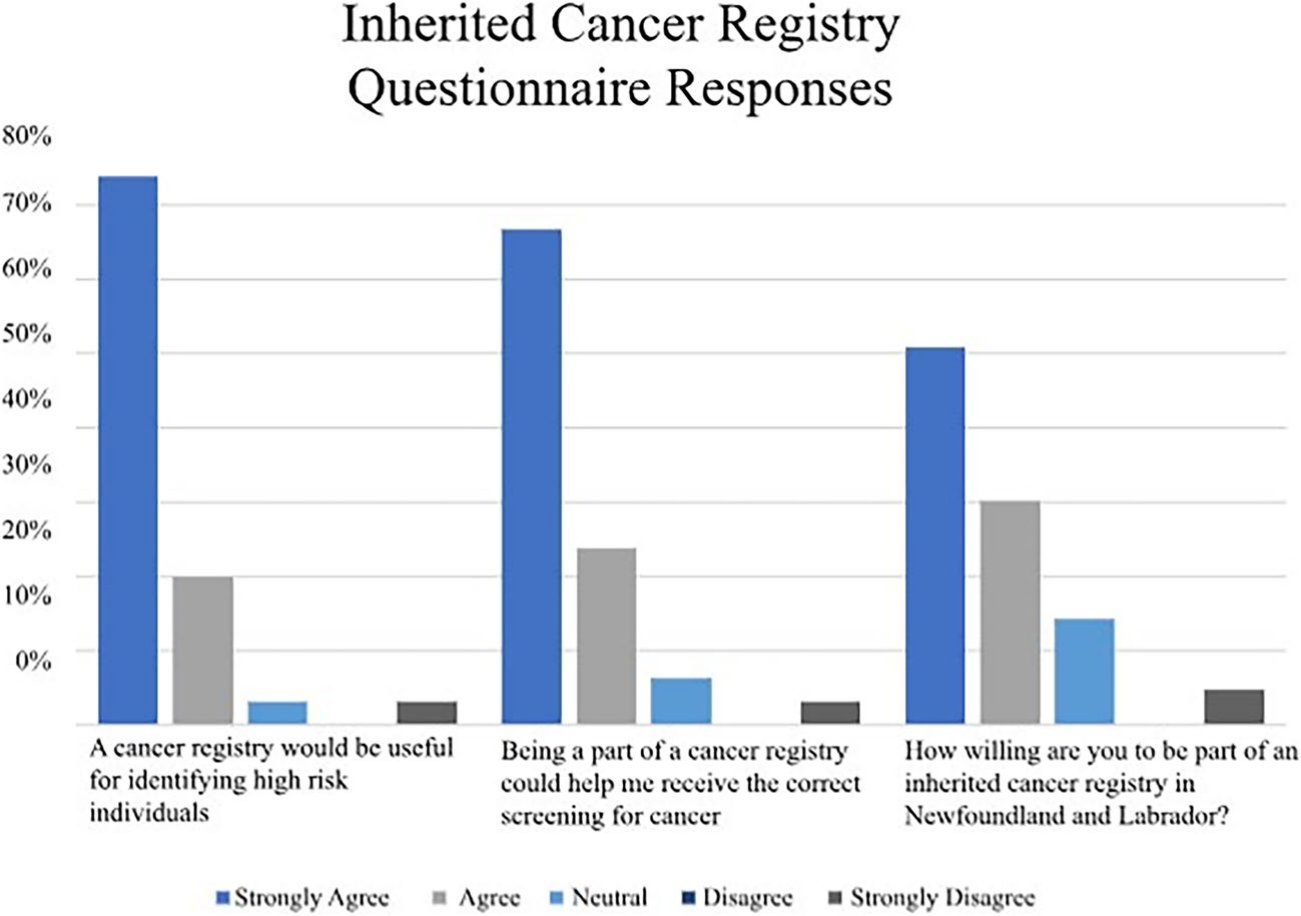
Patient opinions on inherited cancer registry (*n* = 69)

Most patients either agreed or strongly agreed that there were benefits to a cancer registry such as identifying high-risk individuals and ensuring individuals received the correct screening; furthermore, most were willing to be part of such a registry (Fig. 1). In open comments, one patient noted it was “extremely important to have a registry for others to be diagnosed/identified early to provide higher success rates in prevention and treatment.”

Almost two-thirds of respondents (65.2%) agreed or strongly agreed with an additional survey item “I would like reminders about what screening or prevention I could be doing.”

**Fig. 2:**
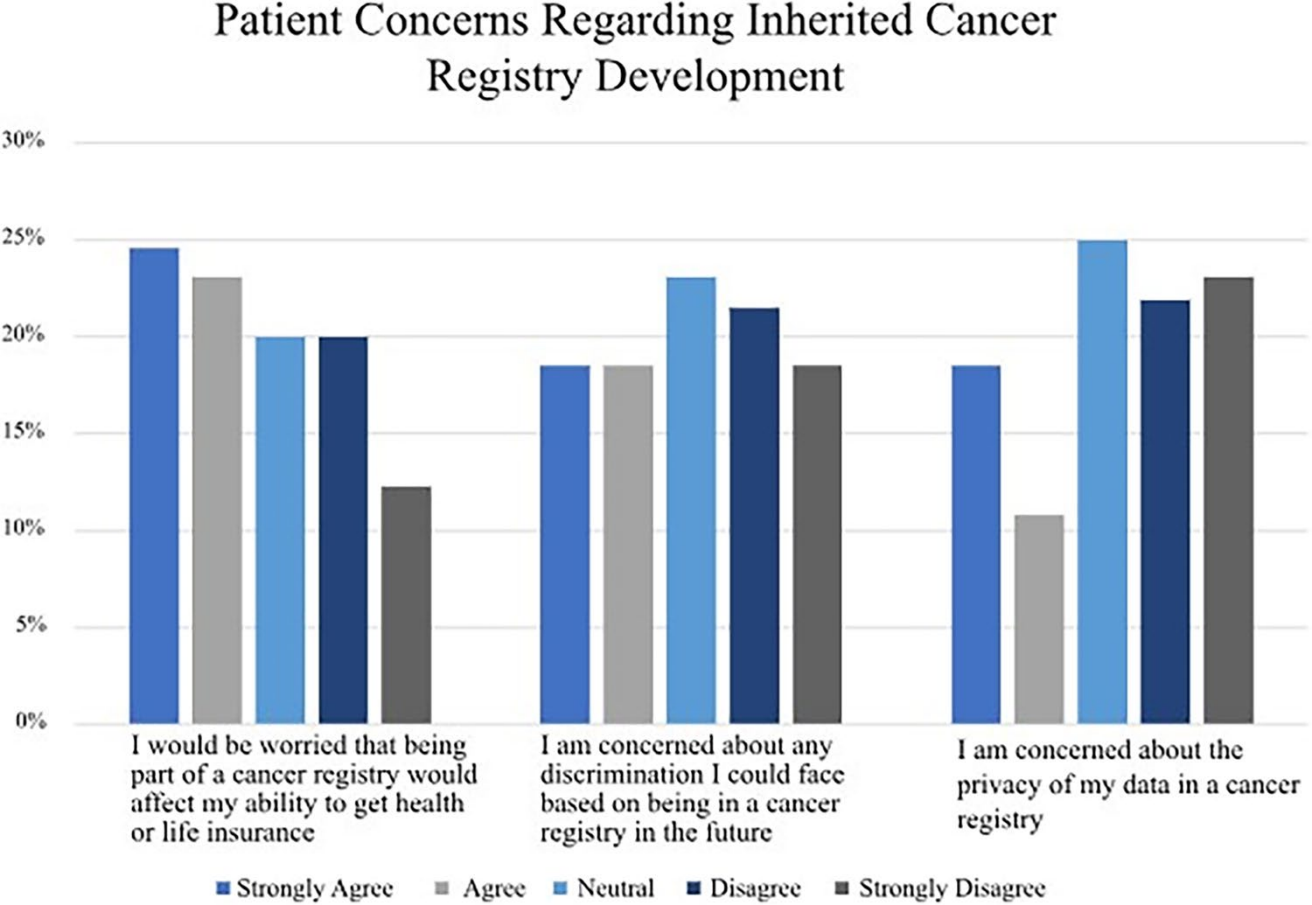
Patient concerns regarding inherited cancer registry (*n* = 69)

Responses to questions related to concerns about the development of an inherited cancer registry were variable (Fig. 2).

About a quarter of respondents expressed concerned over the privacy of their data in a registry, with similar numbers expressing concern about possible discrimination, including in insurance contexts. One survey respondent commented that she would agree with a registry “if controls were put in place for privacy and to prevent insurance coverage or life insurance issues from happening.”

### Qualitative findings

Qualitative data also revealed strong support from participants for the creation of a provincial inherited cancer registry. All 15 interviewees supported creating a provincial inherited cancer registry, and all were willing to take part. Participants noted several positive benefits to such a registry, including not feeling alone and providing a system for reminding patients about upcoming screening and appointments, in line with 65% of respondents who agreed with the survey item about screening reminders. Participants also supported a centralized healthcare service that would monitor their information (see Table 3 for illustrative quotes). This is in line with agreement with survey items measuring the benefit of a registry for identifying high-risk individuals and ensuring they receive appropriate cancer screening (Fig. 1).

**Table 3:** Key themes raised during patient interviews

Theme	Illustrative quotes
The need for a centralized system of inherited cancer care	“It felt like there was too many arms of this that weren’t coming together. It felt like you either had to deal with a genetic doctor, or you had to deal with a breast doctor, or you had to deal with an ovarian [doctor] … it felt like too many of those were off on a tangent, that they weren’t together. There should be one spot that you can go to and someone is there for counseling saying, this is what’s available…” – Patient 13 “Say I get an MRI. Ok I get it done, I don’t know if someone received it or not. I don’t know if someone notices that I haven’t had a mammogram in 8 or 9 months. Should I call someone and ask them if they have track of that, you know? Is someone noticing that I don’t get a mammogram when I need to get it, because I might not necessarily notice myself…but I have a really good gynecologist who orders my appointments too … She knows all about *BRCA* as well, which is really helpful. I’ve spoken to her a couple of times and said, “I haven’t got anything in the mail in a long time I don’t know if I’m overdue, so she has ordered. She has ordered the last few MRIs for me, so I’m not on my own.” – Patient 8 “Like I said, I feel like all the doctors are off in different corridors. There is no central registry, that’s what it feels like.” – Patient 13
Coordinating inherited cancer care through registries	“There’s too many people think that they’re alone. They think that it’s only them, or it’s only their family. If they understood that there was more in it, if there were more doctors that like, when they went with a cancer and realized that in the registry your family is in this, like have you been tested? … They don’t, there’s too many doctors [that] don’t know it. So, I’m thinking it would be effective that way.” – Patient 13 “If there is anything I wish that existed here is just simply that reminder phone call. I think anyone who is going to do the screening, is going to do the screening and anyone who is not, is not. At some point, it’s up to the patient who ultimately has to do it. If there was just some annual reminder … there is something about it having that person call you once a year and say, ‘Hey, it’s that time’.” – Patient 3 “Sometimes people… aren’t proactive about it for themselves. So even if somebody was there to, I guess remind them or take the initiative to say you need to get your appointment, get your screening done.” – Patient 4 **“**They need to have someone that knows more about every aspect of it … like a central group that says, if you decide to have your breasts done, we’re going to send you this way, but you could come back to us because we understand … and [if] you want reconstruction, these are the steps that’s going to be involved, because you don’t always get that from the doctor that is just dealing with the breast, you don’t get that part of it. So, if you had this one person, because you’re more comfortable talking to one than going in and talking to 10 different doctors and saying almost the same thing every single [time] … after a while people don’t share information because you’re burnt out with it. So, if you had one spot that you could sort of, it would be in your file … I think there should be something there.” – Patient 13 “If this registry in any way, shape, or form would help, however small or large it might be, we need to do it.” – Patient 1
Access and use of registry data	“I guess sometimes it’s the compiling all the information together, it would mostly be who is looking at it. I mean, I can see the benefits in it, but when you talk about personal information. If it is insurance companies or if they somehow got it, it’s a lot of very personal information. I know it can be beneficial, but it could also somehow be misused and could be used in harmful ways.” – Patient 10 “With the security stuff, with the stuff being leaked out … Too many medical records now being [accessed]”. – Patient 3

There were only two concerns raised by participants when asked about an inherited cancer registry: the privacy of their personal information and who would have access to it and the specific concern of insurance companies gaining access to their information (similar to the open comments left on the survey). Survey items about potential discrimination and access to data by employers or insurers (Fig. 2) also revealed similar participant concerns.

## Discussion

Little research has explored patients’ views about dedicated inherited cancer services and high-risk registries. Our findings reveal high levels of support for such a service, with minimal (though important) patient concerns. Survey and interview data revealed continuity and coordination challenges with the current system of care of high-risk individuals. Respondents suggested an inherited cancer registry would help identify high-risk individuals and provide a centralized system of risk management for identified carriers. Meiser et al. 2022 also reported positive and altruistic attitudes toward registries in their interviews with a small sample of pathogenic carriers.

Systematically identifying and tracking carriers of pathogenic cancer mutations are critical given the availability of effective prevention and surveillance strategies and the increasing use of genetic and genomic testing (Burn et al. 2020; Meiser et al. 2022). Traditional approaches of identifying individuals at risk for hereditary breast-ovarian cancer or Lynch syndrome include family history, ethnicity, or clinical criteria; however, these approaches miss a substantial number of pathogenic carriers, as high as 50% (Mighton et al. 2022). Previous research has highlighted the benefits of a dedicated inherited cancer service and registry, such as increasing screening rates, decreasing mortality, and facilitating timely research access (Barrow et al. 2013; Ghorbanoghli 2018; Vasen et al. 2016). There are recent calls in the Canadian context for new models of inherited cancer care that include a dedicated registry for carriers (Tindale et al. 2022), and registries also provide a mechanism for patient re-contact in the event of variant reclassification or the establishment of new gene-disease associations (Savatt et al. 2021).

These benefits of registries must be balanced about privacy and data access concerns. Similar to our findings, Meiser et al. (2022) reported their respondents recognized the potential for misuse of registry data by employers or insurers and endorsed the need to balance the potential registry benefits with concerns about patient data safety and confidentiality. These concerns have been reported more broadly in our prior work on public attitudes and expectations about genetics research (Etchegary et al. 2013, 2015) and underscore the need for clear and transparent data governance processes in hereditary cancer registries.

The Dutch Lynch syndrome registry demonstrates a successful registry framework (Vasen et al. 2016) and could be a useful model for managing *BRCA* carriers in Canada. This long-running registry focuses on family follow-up, ensuring the quality of surveillance programs, and appropriate clinical management of individuals enrolled in the registry; outcomes include survival benefits and increased screening rates in high-risk patients.

Dedicated inherited cancer services improve referral rates to appropriate care providers, increase uptake of preventive surgeries, and improve cancer risk management (Lobo et al. 2018; Petelin et al. 2020; Pichert et al. 2010). In addition, these registry programs increase the potential for future research studies (Vasen et al. 2016). For example, the initiation of the Breast Cancer Family Registry, a multinational registry located in Canada, the USA, and Australia, has resulted in the launch of over 80 research projects, conferring positive health outcomes to high-risk families (John et al. 2004).

Our findings have clinical and health policy implications. Results revealed that a sizable minority of *BRCA* carriers are not adherent to risk management guidelines. Both survey and interview respondents expressed a desire for a more cohesive and comprehensive management plan for themselves and their family members. Participants believed that being part of an inherited cancer registry would help them receive coordinated, appropriate, and timely care and risk management services that could ultimately help prevent cancer.

Only a minority of interviewees raised concerns about insurance coverage and potential information leaks with joining a registry, although a greater number of survey respondents expressed such concerns. These results suggest limited awareness about Canadian legislation, specifically Bill S-201 Genetic Non-Discrimination Act, which prohibits and prevents genetic discrimination (Legislative Summary of Bill S-201, 2022). These sorts of concerns have been reported more broadly about the use and access of genetic and genomic data (Etchegary et al. 2022; Genetic Alliance UK, 2015) and suggest that transparent and accessible public and provider education about data storage, usage, and access will be critically important components in designing a provincial inherited cancer service and registry.

### Strengths

This study utilized two data collection methods, interviews and surveys, and captured *BRCA* carriers with a wide range of demographic and clinical characteristics. The population interviewed varied with regard to their age, location, personal history of cancer, and prophylactic surgeries. The survey had a reasonable response rate of ~50%.

In our study setting, this population has not been studied in great depth; thus, this project’s information provides previously undocumented information about this group of patients. This population is a complete population-based cohort, with all-female *BRCA* carriers in the province identified by central cancer care, central genetics services, and province-wide electronic medical records. The inclusion of patient partners in the study is a strength, contributing to capacity building in patient-oriented research and ensuring the study aligned with the priorities and concerns of patients.

### Limitations

The number of study participants remains relatively small; study findings are based on individuals living in one province, and thus, the information presented may not be generalizable to other populations. Nonetheless, study findings are similar to the wider literature. We purposefully focused on female *BRCA* mutation carriers because they represent (by far) the most significant percentage of carriers in this jurisdiction. We recognize that *BRCA* risk management is also relevant to men, but we cannot speak to the male carrier experience through this study. We were unable to link 25 survey respondents with their clinical and demographic information and unable to correctly compare survey respondents with non-respondents. This is a clear limitation; however, given that this work has not been undertaken with the *BRCA* population in the province previously, survey results have descriptive value. The survey used was not a validated questionnaire; instead, it comprised descriptive questions to gather a broad picture of *BRCA* mutation carriers’ experiences in the province. However, as this was a patient-oriented research project, patient study team members reviewed all study instruments and did not note difficulties with item comprehension.

The focus of this project was on patient attitudes and concerns regarding inherited cancer registries. However, creating and maintaining such a registry are dependent not only on patient support and enrolment but also a host of other considerations such as dedicated funding, collaboration with a variety of agencies and organizations to acquire registry data, and creating data compatibility standards for software and datasets across data custodians (e.g., Meiser et al. 2022). Future research on these registry inputs and processes are necessary, in tandem with patient attitude research such as presented here.

## Conclusions

Findings from this study revealed that female *BRCA* carriers support a coordinated care registry for individuals with inherited cancer risks. These individuals believe such a registry could help improve the coordination of burdensome, life-long risk management and help identify at-risk individuals not yet tested. However, important patient concerns about protecting their privacy and their health data confidentiality must be addressed in any patient and public information documents about a registry. Findings can be used to inform the planning and implementation of a provincial inherited cancer registry and add to the evidence base on how best to provide care to carriers of inherited cancer mutations. We recommend that such a program include patient education, electronic management of patient surveillance and surgeries, family physician outreach and support, assistance with at-risk relative recruitment, opportunities for engagement with research, and ongoing quality assurance.

### Supplementary information

ESM 1 (DOCX 17 kb)

ESM 2 (DOCX 14 kb)

## Data Availability

A copy of the descriptive survey and interview guide is included in Supplementary files.
