# Supplementary material for: “There should be one spot that you can go:” BRCA mutation carriers’ perspectives on cancer risk management and a hereditary cancer registry
Source: J Community Genet. 2023 Oct 21;15(1):49–58. doi: 10.1007/s12687-023-00685-5 (PMC10858006; doi:10.1007/s12687-023-00685-5)
Supplement: Supplementary file 1 — (DOCX 17 kb) [file 12687_2023_685_MOESM1_ESM.docx]

Descriptive Survey BRCA study

The purpose of this survey is to learn about feelings people have when they know that they have a disease-related gene. You are being asked to fill out this survey because you have tested positive for (or have recently learned that you carry) a gene that may be associated with an increased risk of cancer. All of your answers are confidential. There are no right or wrong answers, and people vary widely in their responses to these questions. If there is anything that you are not comfortable answering, please leave it blank and indicate that you do not wish to answer. (Items in this section were collected largely for quality improvement purposes at the inherited cancer prevention clinic and are not published).

| **Please read each item, then circle the number in the box with the response that best describes your feelings at this time.** | **Strongly Agree** | **Agree** | **Neutral** | **Disagree** | **Strongly Disagree** | **Not Applicable** |
| --- | --- | --- | --- | --- | --- | --- |
| 1. I felt that I received clear information about the results of my genetic testing | 1 | 2 | 3 | 4 | 5 | 6 |
| 2. My family doctor was knowledgeable about BRCA mutations | 1 | 2 | 3 | 4 | 5 | 6 |
| 3. The guidelines for screening were clear | 1 | 2 | 3 | 4 | 5 | 6 |
| 4. I understand my risk of breast cancer | 1 | 2 | 3 | 4 | 5 | 6 |
| 5. I understand my risk of ovarian cancer | 1 | 2 | 3 | 4 | 5 | 6 |
| 6. I understand my options for preventative ovarian surgery | 1 | 2 | 3 | 4 | 5 | 6 |
| 7. I understand my options for breast cancer prevention | 1 | 2 | 3 | 4 | 5 | 6 |
| 8. I understand the pros and cons of preventative mastectomy | 1 | 2 | 3 | 4 | 5 | 6 |
| 9. I understand the pros and cons of preventative ovarian surgery | 1 | 2 | 3 | 4 | 5 | 6 |
| 10. I have had preventative ovarian surgery | 1 | 2 | 3 | 4 | 5 | 6 |
| 11. If you decided not to have preventative ovarian surgery, the reason why at this time. Please circle all that apply:   1. No doctor has recommended surgery 2. I am too young 3. I want to have more children 4. Afraid of menopause 5. Surgical Risks 6. Arranging time off of work 7. Childcare arrangements 8. Other surgeries required 9. Recovering from Cancer 10. Other________________________________ |  |  |  |  |  |  |
| 12. I was able to book breast MRI appointments | 1 | 2 | 3 | 4 | 5 | 6 |
| 13. I had trouble getting breast MRI appointments | 1 | 2 | 3 | 4 | 5 | 6 |
| 14. If you had trouble, the reason why you had trouble getting breast MRI appointments. Please circle all that apply:   1. Travel 2. Time away from work 3. Childcare arrangements 4. Financial 5. MRI machine restrictions 6. Anxiety/Claustrophobia 7. Other_________________________ |  |  |  |  |  |  |
| 15. I would like reminders about what screening or prevention I could be doing | 1 | 2 | 3 | 4 | 5 | 6 |
| 16. I would like to combine doctor’s appointments and tests | 1 | 2 | 3 | 4 | 5 | 6 |
| 17. I do not know where to get information or support about my personal cancer prevention | 1 | 2 | 3 | 4 | 5 | 6 |
| 18. I feel comfortable telling my relatives about BRCA mutation | 1 | 2 | 3 | 4 | 5 | 6 |
| 19. I feel that all my family members understand about BRCA | 1 | 2 | 3 | 4 | 5 | 6 |
| 20. I wait too long for tests or appointments because of waitlists | 1 | 2 | 3 | 4 | 5 | 6 |

| 21. In what income category would you place your household:   1. Low income 2. Middle income 3. High income |
| --- |
| 22. What is the highest degree or level of education you have completed?   1. Less than high school 2. High school graduate (includes equivalency) 3. Completed post-secondary training 4. Completed university degree 5. Masters or professional degree 6. Ph.D. |

**The next set of questions are about inherited cancer registries: (reported in this paper)**

An inherited cancer registry is basically a database that stores patient medical and personal information, like how old you are, where you live and who your family doctor is, as well as test results. These registries have been shown to help in the ongoing management and clinical care of people affected by BRCA and other inherited cancers. In NL, we do not have a formal inherited cancer registry. Please give us your thoughts on the following questions about inherited cancer registries.

| **Please read each item, then circle the number in the box with the response that best describes your feelings at this time.** | **Strongly Agree** | **Agree** | **Neutral** | **Disagree** | **Strongly Disagree** |
| --- | --- | --- | --- | --- | --- |
| A cancer registry would be useful for identifying high risk individuals. | 1 | 2 | 3 | 4 | 5 |
| Being a part of a cancer registry could help me receive the correct screening for cancer. | 1 | 2 | 3 | 4 | 5 |
| I would be worried that being part of a cancer registry would affect my ability to get health or life insurance in the future. | 1 | 2 | 3 | 4 | 5 |
| I am concerned about any discrimination I could face based on being in a cancer registry. | 1 | 2 | 3 | 4 | 5 |
| I am concerned about the privacy of my data in a cancer registry. | 1 | 2 | 3 | 4 | 5 |
| How willing are you to be part of an inherited cancer registry in Newfoundland and Labrador? | 1 | 2 | 3 | 4 | 5 |
