# Supplementary material for: “There should be one spot that you can go:” BRCA mutation carriers’ perspectives on cancer risk management and a hereditary cancer registry
Source: J Community Genet. 2023 Oct 21;15(1):49–58. doi: 10.1007/s12687-023-00685-5 (PMC10858006; doi:10.1007/s12687-023-00685-5)
Supplement: Supplementary file 2 — (DOCX 14 kb) [file 12687_2023_685_MOESM2_ESM.docx]

**Interview Guide: BRCA Study**

Open-ended questions are designed to elicit commentary on experiences with **cancer in the family** (first awareness of hereditary link, perceived personal risk, screening motivation) and **genetic testing** (decision-making, counseling experiences, reaction to status, understanding implications, impact on family). Additional questions evolve from adjusting to carrier status, screening experiences, and health care service needs.

**1. Please tell me about your family’s experience with cancer.**

| **Questions:** | **Prompts:** |
| --- | --- |
| Can you tell me about your family history of cancer? | When do you recall hearing about BRCA? How did you find out about BRCA? |
| Can you tell me how you came to discover you personally were at risk for BRCA? | How was this explained to you?  By whom? |
| Can you tell me about your genetic testing experience? | Who recommended genetic testing?  Did you understand the implications of BRCA for your health? For your family?  Did you feel satisfied with the level of information you received? |

**2. Can we talk about what having BRCA means for your health (and healthcare).**

| **Questions:** | **Prompts:** |
| --- | --- |
| What recommendations were given to you after being diagnosed with BRCA? | Was any kind of screening recommended? Was genetic testing of other relatives recommended? |
| What benefits of preventative gynecologic surgery were explained to you? | What disadvantages were explained to you? |

**3. I would like to talk about how you manage your cancer risk.**

| **Questions:** | **Prompts:** |
| --- | --- |
| What kinds of things have you been doing to manage your cancer risk? | Do you do any regular screening like mammograms or MRIs? |
| Have you had preventative surgery (e.g., gynecological or mastectomy?) | What was your experience like when trying to make that decision about surgery? Was this an easy decision to make? |
| Did you feel you had enough information to make an informed decision about how you have decided to manage your cancer risk? | Have you ever regretted your decision?  Is there anything you wish you would have done differently with regards to making decision about cancer risk management? |

4. **Tell me about life with BRCA.**

| **Questions:** | **Prompts:** |
| --- | --- |
| How would you describe your quality of life today? | Is it hard to manage your screening?  Are you worried about the risk of cancer?  How do you feel BRCA has impacted your family? |

**5. Healthcare needs**

| **Questions:** | **Prompts:** |
| --- | --- |
| Have there been any barriers to receiving the screening that has been recommended to you? | Is there anything you feel you need in relation to your BRCA status, but have difficulty accessing? |

**6. Inherited cancer registries (focus of this paper)**

“An inherited cancer registry is basically a database that stores medical and personal information, like how old you are, where you live and who your family doctor is, as well as test results. These registries have been shown to help in the ongoing management and clinical care of people affected by BRCA and other inherited cancers. In NL, we do not have a formal inherited cancer registry.”

| **Questions:** | **Prompts:** |
| --- | --- |
| If an inherited registry were set up, do you think you would agree to take part? |  |
| What sorts of benefits and risks come to mind if our province set up an inherited cancer registry? | What do you think the advantages of a registry would be?  Do you have any concerns? |

**Please feel free to share any other thoughts you have on living with BRCA.**

**Is there something you would like to share that we have not discussed in our interview?**

**Thank you very much for taking the time to talk with me today. Please touch base anytime with any other questions or concerns.**
